# The Role of Local and Upstream Colonisation in Determining Stream Periphyton Metacommunity Assemblages

**DOI:** 10.1002/ece3.70850

**Published:** 2025-01-17

**Authors:** Daniel Zamorano, Travis Ingram, Christoph D. Matthaei

**Affiliations:** ^1^ Department of Zoology University of Otago Dunedin New Zealand

**Keywords:** dispersal processes, drift community, field experiment, functional guilds, mass effect, New Zealand

## Abstract

Stream periphyton is an ideal study system for explaining how dispersal shapes community patterns. Few studies have tried to investigate periphyton metacommunities at the reach scale, and studies comparing local versus upstream periphyton propagule sources are lacking. We aimed to address these knowledge gaps by disentangling environmental constraints and dispersal sources, including dispersal hypotheses related to periphyton functional guilds. We covered 25‐m sections of streambed with plastic silage cover sheets in three streams in Southern New Zealand, allowing river water to flow over the sheets. Samples on top of these sheets allowed periphyton colonisation only by drifting upstream propagules, while ‘control’ samples placed directly upstream of the plastic sheets were colonised by local and upstream propagules. We collected samples after 7, 14, and 25 days of colonisation. Response variables included periphyton biomass, community structure, and relative abundances of functional guilds. Control samples showed 1.5–6 times higher cell densities than plastic‐cover samples, suggesting that local colonisation is very important for biomass accrual. Periphyton communities on both tile types became more similar to each other with time, indicating that environmental filters overcame effects of colonisation sources. While motile and flagellated taxa showed the ability to reach their preferred microhabitats in all streams, the responses of the remaining functional guilds did not follow the expected patterns. We conclude that periphyton community assembly strongly depends on reach‐scale connectivity, which results in higher biomass accrual and community structure. These findings suggest that the mass effect paradigm is likely to be the principal metacommunity process shaping stream periphyton communities at the reach scale.

## Graphical abstract

Field experiment conducted to determine the role of local and upstream propagules as drivers of stream periphyton communities. Community composition, structure, and functional guilds were assessed. Local colonisation was identified as the primary propagule source, leading to a mass effect.

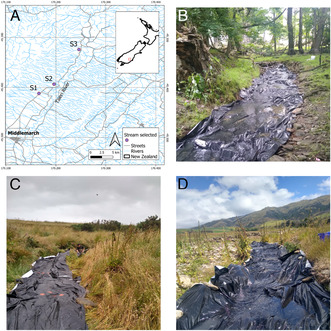

## 1 Introduction

A metacommunity is a set of local communities that are linked by the dispersal of multiple interacting species (Leibold et al. 2004; Wilson 1992). Community structuring depends on broadly defined processes (Lortie et al. 2004): stochasticity, colonisation, migration, abiotic conditions, competition, facilitation, and predation. During past decades, community ecology research focused mainly on biotic interactions such as competition and predation, and the role of abiotic conditions shaping local community structures (Vellend 2010). However, the metacommunity framework has revitalised the study of migration and colonisation, incorporating dispersal constraints as a community driver similarly important as environmental selective strength and demonstrating the role of regional processes in modulating local communities (Lindström and Langenheder 2012; Vellend 2010). Together with new technologies to track species movements, such as GPS, radio‐tracking, and remote sensing, the last 20 years have brought in a new era of research on species migration and its role in modulating communities (Driscoll et al. 2014).

This paradigm shift towards increased focus on dispersal processes has also occurred in studies of the specific metacommunities made up of periphyton in streams. There is a long tradition of research on freshwater periphyton communities and their relationship with environmental variables (McIntire 1968; Pringle et al. 1988; Shelford and Eddy 1929), but the traditional view that microorganisms are ubiquitously distributed (Baas‐Becking 1934) postponed studies on their dispersal processes. However, more recently, dispersal processes of stream periphyton have received more attention, after the realisation that principles of spatial and landscape ecology can also be applied to microbial communities (Larned 2010; Martiny et al. 2006).

At the catchment and regional scales, a number of studies have investigated environmental versus dispersal constraints using large, survey‐based datasets, and the findings of these studies support the idea that dispersal processes modulate periphyton communities (Besemer et al. 2012, 2013; Bottin et al. 2016; Leboucher et al. 2020; Soininen et al. 2016). By contrast, fewer studies have focused on the role of periphyton dispersal processes at smaller spatial scales. In fluvial geomorphology, the reach scale is the spatial unit longer than a site (> 10 m) and shorter than a segment (< 1 km). A reach is defined as a river section (~ 100 m) lying between breaks in channel slope and local side slopes, with its length depending on river morphology (Domisch et al. 2015; Frissell et al. 1986). The reach scale is the most frequently used scale to define sampling units in ecological studies because it allows for the simultaneous assessment of biological communities and physical riverine properties (Frissell et al. 1986; Kuemmerlen et al. 2019). However, most studies aimed at understanding periphyton colonisation at the reach scale have been developed in the context of drought, assessing biofilm accrual after flow resumption when permanent pools and dry biofilms were present in the investigated reaches (Robson 2000; Robson et al. 2008; Robson and Matthews 2004).

The remaining studies on periphyton dispersal processes have been conducted at smaller spatial scales. At the site scale (< 10 m), studies have focused on biofilm accrual and community composition pre‐ and post‐flood disturbance, rather than directly studying dispersal processes (Matthaei, Guggelberger, and Huber 2003; Peterson et al. 1994; Peterson 1996a). At the microhabitat scale (< 10 cm), a few studies have shown that the immigration rate is more important than the reproduction rate for determining periphyton community composition during the early stages of biofilm accrual (Hödl et al. 2014; McCormick 1991; Stevenson 1984). Further, it has been observed that higher flow velocity (and similar hydraulic variables) increases periphyton immigration rates (McCormick 1991; Stevenson 1983; Woodcock et al. 2013).

Periphyton species exhibit a wide range of dispersal mechanisms, each of them interacting with several environmental contexts and having an impact at different spatial scales. Some periphyton species show high motility at the microhabitat scale, supporting the idea of frequent colonisation from near‐streambed habitats. For example, flagellates exhibit free motility (Johnson, Tuchman, and Peterson 1997), and some diatoms can move across surfaces by gliding (Poulsen et al. 2022). Periphyton motility responds to environmental physical and chemical conditions, such as light, temperature, salinity, desiccation, pH, and nutrients (Serôdio 2021), allowing periphyton species to actively seek out resources and colonise adjacent habitats. Other functional traits of periphyton species also enhance their dispersal capabilities. Filamentous life forms, unicellular taxa, and taxa unattached to substrata show a higher possibility of being dragged by the current (Biggs, Stevenson, and Lowe 1998; Steinman 1996), thus they should express a higher dispersal capability. Periphyton taxa possessing traits related to higher dispersal capability are expected to be more prevalent in early‐stage biofilms because of their fast colonisation (Biggs, Stevenson, and Lowe 1998; Passy and Larson 2011). The opposite is expected for colonial taxa because their morphology should result in faster sinking rates and thus lower dispersal capability (Borics et al. 2023). Therefore, it is necessary to consider successional time together with functional guilds to describe periphyton dispersal processes.

High connectivity at the catchment scale between periphyton communities suggests that flow‐mediated dispersal processes of periphyton species are due to drag by river flow (Besemer et al. 2012, 2013; Graco‐Roza et al. 2020; Kärnä et al. 2015). While the mechanism by which upstream colonists immigrate to downstream habitats is not clear, it has been established that there is a relationship between the upstream community in the drift (hereafter, drift community), the periphyton taxon pool found in river‐water samples, and the periphyton community on the streambed. For example, taxon richness and density in periphyton and drift community have been found to be correlated (Robson et al. 2008; Roeder 1977), and biofilm biomass accrual is related to drift colonisation rates (Peterson 1996a; Stevenson and Peterson 1991). Yet, several studies found that both communities were different when compared against young (3 days old) (Peterson 1996a) or mature river‐bed biofilms (3 weeks) (Besemer et al. 2012; Peterson et al. 1994), thus making it challenging to find consistent similarities between both. Overall, the existing evidence suggests that, while the drift community is an important source of propagules, its relationship with the periphyton community is still unclear.

When attempting to disentangle dispersal processes in periphyton communities, one needs to consider the different spatial scales involved (propagules from adjacent microhabitats, upstream reaches, upstream rivers, and distant catchments), the varied dispersal mechanisms, and the interactions with the environmental context and successional time. Given this complexity, we suggest that a prudent starting point is to differentiate between propagule sources by splitting them into two categories: local and upstream (Robson 2000; Robson et al. 2008; Robson and Matthews 2004). By investigating both these propagule sources at the reach scale, the present study aims to address the knowledge gaps highlighted in the previous paragraphs. At the catchment scale, long‐distance dispersal processes become more relevant for maintaining community connectivity, whereas at the microhabitat scale, colonisation from the nearby streambed is likely to be the principal source of propagules, and long‐distance colonists are considered exceptional. It is at the reach scale where both propagule sources interact, elucidating how different dispersal processes shape metacommunity assembly.

We developed an in‐stream experiment in which we investigated colonisation dynamics of benthic periphyton communities under two contrasting dispersal scenarios: tiles exposed to local and upstream colonisation (control tiles), and tiles exposed in the same rivers but cut off from local colonisation from the river‐bed using a large plastic‐cover sheet (plastic‐cover tiles). Based on a review of the related literature, we hypothesised that: (H1) plastic‐cover tiles will develop biofilms more slowly because colonisation rates will be lower; (H2) a higher proportion of functional guilds with high dispersal capabilities will colonise plastic‐cover tiles because these guilds can overcome the barrier imposed by the plastic sheet; and (H3) the community developing on plastic‐cover tiles will be more similar to the drift periphyton community than to the control‐tiles community because colonising species will come exclusively from further upstream. To our knowledge, no previous research has investigated the impact of these periphyton traits on habitat colonisation at the reach scale.

## 2 Methods

### 2.1 Study Sites and Experimental Design

**FIGURE 1:**
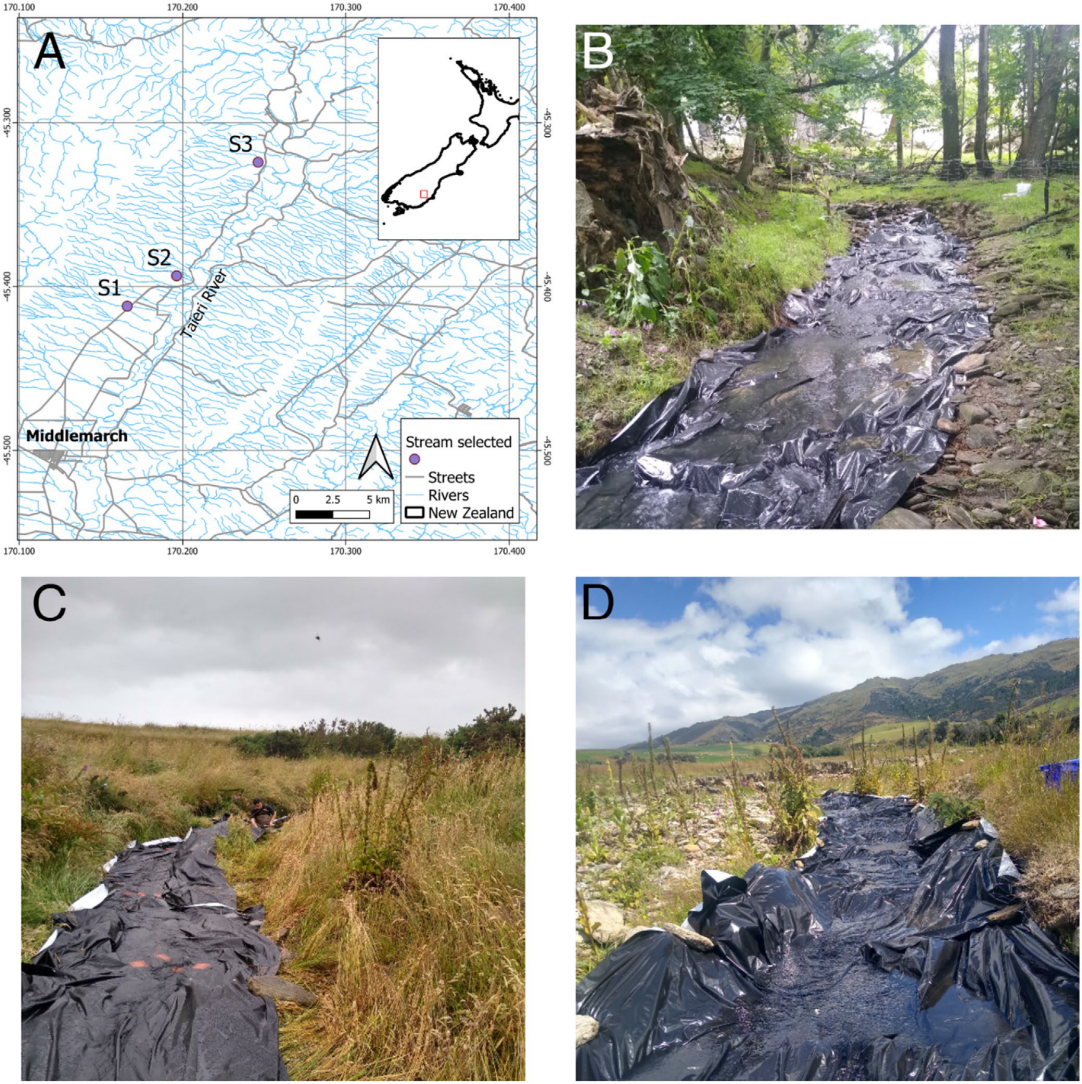
Map of the study area (A). Reaches of Stream 1 (B), Stream 2 (C), and Stream 3 (D) where the plastic‐cover treatment was implemented.

The study was carried out in three 3rd‐order streams near the town of Middlemarch, in the Otago region of the South Island of New Zealand (Figure 1). The streams are situated close to each other (within ~10 km) and drain the same geological formation, the Rock and Pillar Range (maximum elevation 1450 m a.s.l.). They are characterised by gravel and cobble substrata, and their flow is highly dependent on snowmelt and rainfall in this semi‐arid region (Raab et al. 2022), which has an average annual rainfall of ~650 mm (Tait, Macara, and Paul 2014). The experiment was carried out in February 2023 (Austral summer). The studied section of Wandle Creek (S1) is 2 m wide and well‐shaded, with a high percentage of riparian tree cover and the presence of some fine sediment on the bed surface. House Creek (S2) is the smallest stream (~1 m width), and the catchment surrounding and upstream of the study reach is covered in native tussock grassland. The studied section of Scrub Burn Creek (S3) is a stony‐bottomed reach surrounded by livestock pasture (sheep and beef grazing), with native tussock grassland further upstream. While this stream is medium‐sized (~4 m wide), our experiment was placed in a stream branch of ~1.5 m width.

We defined propagules from biofilms on the nearby streambed surface as local colonists and drifting propagules from the upstream streambed as upstream colonists. Local colonists embody short‐distance dispersal processes, while upstream colonists embody long‐distance, flow‐mediated dispersal processes. In each stream, we identified a 50‐m reach with homogeneous morphological conditions. In these sections, we exposed ceramic tiles (10 × 10 × 1 cm) under two experimental scenarios: normal colonisation (control tiles) and colonisation only from upstream (plastic‐cover tiles). The latter scenario was implemented by covering the entire wetted width of the streambed surface of the downstream half of the reach (25 m) with silage cover (Silage Cover Black/White, Donaghys Ltd., Christchurch, New Zealand) (Figure 1), a sturdy plastic sheet (black on one side, white on the other) commonly used to cover silage and crops on farms. The silage cover (with the black side up) was attached to the streambed using tent pegs and metal fence posts laid down flat on the bed surface. The plastic sheet also covered about 50 cm of each stream bank outside the wetted width. This plastic cover allowed river water to flow over it while preventing the tiles placed on top of it from being in contact with the streambed, allowing periphyton colonisation only via drift from upstream. For the scenario of normal colonisation, tiles were placed directly onto the 25‐m stretch of streambed directly upstream from where the silage cover was attached.

Tiles were sampled on the 7th, 14th, and 25th days after the experiment started (hereafter d7, d14, and d25, respectively). Tiles were arranged in replicated groups of four tiles each, one per sampling date plus an extra tile. Each tile group was attached to the streambed by tent pegs to prevent them from getting washed away during periods of elevated discharge. Each tile group was placed 3 m apart from other groups and at least 5 m away from the upstream and downstream edges of the plastic sheet, and thus the nearest periphyton‐covered streambed surfaces. In each stream, we exposed 40 tiles in total (2 treatments × 3 sampling periods × 6 replicates = 36 tiles) in 10 tile groups, with 2 surplus tiles in one of the tile groups. Benthic periphyton sampling was complemented by sampling the drift community. For this purpose, five replicates of 250 mL of stream water per experimental treatment were collected on each sampling date, with each sample being collected 5 m apart, moving upstream.

Regrettably, the studied reach in S3 dried up after the first sampling date due to dry weather and high local streambed porosity, resulting in the loss of one replicate per treatment on Day 7, and all samples for Day 14 and Day 25. Moreover, the studied reach in S2 was vandalised before the second sampling date, resulting in the loss of four replicates in this stream on Day 14, and of all samples for Day 25. These events also prevented us from characterising microhabitat conditions in these streams, and drift‐water samples were collected only in S1 and S2. Consequently, S1 was the only stream in which the entire planned dataset could be collected. To slightly increase our database, the four additional tiles exposed in S1 (see above) were included as Day 25 samples. Therefore, our final dataset included 69 tile samples (S1: Day 7 = 12, Day 14 = 12, Day 25 = 16; S2: Day 7 = 12, Day 14 = 7; S3: Day 7 = 10) and 15 drift‐water samples (S1 = 10, S2 = 5).

### 2.2 Laboratory Procedures

For each tile, all periphyton on the top surface was scraped off with a toothbrush into a tray. The resulting slurry was rinsed into Falcon tubes and topped up with deionised water to 55 mL. This sample was divided into two parts: 10 mL were preserved for future algal identification by adding Lugol's iodine, and 45 mL were used to estimate ash‐free dry mass (AFDM) as a proxy of biofilm biomass. For AFDM, samples were dried at 105°C for 24 h, weighed in an analytical balance, ashed at 400°C for 4 h, and then weighed again. AFDM procedures and calculations were carried out following (Biggs and Kilroy 2000).

We enumerated at least 200 algal or cyanobacterial cells per sample by examining 8–30 microscope fields at 400x magnification under an inverted microscope (Zeiss Axiovert 25, Jena, Germany). Each inspected field was checked at 1000x magnification to confirm all classifications. Taxa were identified to species for diatoms and to genus for cyanobacteria and green algae using standard keys (Bellinger and Sigee 2010; Biggs and Kilroy 2000; Entwisle, Sonneman, and Lewis 1997; Foged 1979; Jüttner et al. 2023; Moore 2000; Spaulding et al. 2021). Filamentous or colonial taxa were treated as “natural counting units”, by counting as one unit each filament or algal colony found in a sample (Charles, Knowles, and Davis 2002). These units are referred to as “cells” henceforth.

### 2.3 Response Variables

All data analyses and plots were carried out in R version 4.3.1 (R Core Team 2023). A list of all response variables, each with a brief description, the specific hypothesis tested, and details regarding transformations, is provided in Table 1. We estimated Taxon Richness and the Shannon‐Wiener diversity index (hereafter: Hill‐Shannon index) using the function iNEXT from the R package iNEXT (Hsieh, Ma, and Chao 2016). This function estimates Hill numbers (Chao et al. 2014), or the effective number of taxa, by correcting and extrapolating them as a function of sample size, thus allowing us to standardise for sampling effort. Richness and Hill‐Shannon values were estimated with an endpoint at 300 and using 999 replications. Further, the Berger‐Parker Dominance index (BP Dominance index), based on the proportional abundance of the most common taxon (Berger and Parker 1970; Guevara, Hartmann, and Mendoza 2016), was estimated using the function diversity from the R package diverse.

**TABLE 1:** Summary of periphyton response variables, organised by hypotheses tested.

Response variable	Variable category	Details	For	Hypothesis related	Transformation
Biomass	Periphyton abundance	Ash‐free dry mass (AFDM)	H1	Higher Biomass is expected in PC samples due to higher colonisation from the nearby streambed	Ln‐transformed
Cell density		Total cell density		Higher Cell Density is expected in PC samples due to higher colonisation from the nearby streambed	Ln‐transformed
Richness	Community diversity	Taxon richness corrected by sample size			Raw data
Cyanobacteria	Algal‐division guilds		H2		Ln‐transformed
Green algae		The model's singularity led us to remove the random factor and the interaction of Time × Treatment, due to the low variance explained by both			OrdNorm transformed (Peterson 2018)
Diatom					Raw data
Colonial	Life‐form guilds			Complex life form implies shorter travel distances. Higher density expected in CT samples (Borics et al. 2023)	Raw data
Filamentous				Long‐shape life form suggests higher probability of being dragged by flow. Higher density expected in PC samples (Biggs, Stevenson, and Lowe 1998; Steinman 1996)	Ln‐transformed
Flagellates				Free motility. Higher density expected in PC samples (Johnson, Tuchman, and Peterson 1997)	Ln‐transformed
Unicellular		Unicellular and non‐flagellate taxa		Small size suggests higher probability of being dragged by flow. Higher density expected in PC samples (Biggs, Stevenson, and Lowe 1998; Steinman 1996)	Ln‐transformed
Low	Attachment‐to‐Substrate guilds	Algae with no fixation structures such as flagellates or entangled filaments		Higher probability of being dragged by flow. Higher density expected in PC samples (Biggs, Stevenson, and Lowe 1998; Steinman 1996)	Raw data
Medium		Erect diatoms and filamentous forms with pad/stalk		Higher position within the biofilm mat suggests higher probability of being dragged by flow. Higher density expected in PC samples (Biggs, Stevenson, and Lowe 1998; Steinman 1996)	Ln‐transformed
High		Adnate and prostate diatoms		Lower probability of being dragged by flow. Higher density expected in CT samples	Ln‐transformed
Non‐motile	Motility guilds	Algae attached to substrate with no motility		Lower dispersal capabilities. Higher density expected in CT samples	Raw data
Gliding		Diatoms able to move by gliding on surfaces		Higher dispersal capabilities. Higher density expected in PC samples (Lange, Townsend, and Matthaei 2016; Passy 2007; Poulsen et al. 2022)	Raw data
Drifting‐motile		Algae with no attachment capability which are constantly dispersed by the river flow		Higher dispersal capabilities. Higher density expected in PC samples (Lange, Townsend, and Matthaei 2016; Passy 2007)	Raw data
Hill‐Shannon index	Community structure	Classic Shannon‐Wiener diversity index corrected by sample size. Based on taxon richness and evenness	H3		Raw data
BP Dominance index		Proportion of the most abundant taxa			Binomial distribution used due to range between 0 and 1. No ES estimated due to lack of comparable methodology with LMMs

*Note:* Columns: Variable category, community pattern characterised by each variable; Details = relevant details of variable; For = hypotheses for which the response variable is relevant; Hypothesis related = hypothesis tested for each response variable including relevant references, transformation = specific transformation applied before analysis.

Abbreviations: CT, control tiles; PC, plastic‐cover tiles.

Periphyton functional composition was investigated by evaluating the presence of a variety of morphological and behavioural algal taxon traits per sample, using as a reference the trait‐based framework proposed by Lange, Townsend, and Matthaei (2016). Each taxon recorded was classified using four functional guild classes: algal division provides information about environmental conditions (Biggs, Stevenson, and Lowe 1998; Ferragut and de Campos Bicudo 2010), life form and attachment to substrate are related to the periphyton successional state and to the probability of being dragged by the river flow (e.g., prostrate taxa are described as early colonisers) (Biggs, Stevenson, and Lowe 1998; Lange, Townsend, and Matthaei 2016; Stevenson et al. 1996), and motility is a trait related to dispersal capabilities and habitat colonisation (Lange, Townsend, and Matthaei 2016) (Table 1). Red and yellow algae were rare in our samples and therefore not included in the statistical analysis. We calculated relative density for each functional guild in each sample.

### 2.4 Statistical Analysis

To evaluate periphyton accrual over time and between treatments (H1), we assessed the response variables biomass, taxa richness, and cell density. Changes in functional guilds were also assessed over time and between treatments (H2). To compare the periphyton community against upstream propagules (H3), we incorporated Drift Water (DW) as a treatment category. We compared communities assessing changes in Hill‐Shannon diversity index and BP Dominance index while we performed a permutational multivariate analysis of variance (PERMANOVA) to compare community assemblages. We carried out linear mixed‐effects models (LMMs) for all individual response variables. The interaction between time and treatments was nested within the sampled streams, controlling for the unequal sample sizes determined by the external circumstances explained above. All LMMs included tile groups as a random factor. LMMs were performed using the function lmer from the lme4 package (Bates et al. 2015).

All model results were complemented by determining effect sizes (ES) for each predictor variable and by applying posterior pairwise post hoc analyses. We considered results as significant when the p‐value was significant (at α = 0.05) and effect size was at least biologically relevant (> 0.10) (Nakagawa and Cuthill 2007), or when the *p*‐value was near‐significant (0.05 < *p* < 0.1) and the effect size was > 0.20. For LMMs, ES was obtained from partial ω^2^ values, estimated using the effectsize package (Ben‐Shachar, Lüdecke, and Makowski 2020). For LMMs with significant results, pairwise post hoc tests were carried out using the function emmeans from the emmeans package (Lenth 2023).

PERMANOVA was performed using the function adonis2 in the vegan package (Oksanen et al. 2015). The community matrix was transformed to relative cell density per sample to enable comparison of different cell density units between drift samples (cells/mL) and periphyton samples (cells/cm^2^). Then, the matrix was log‐transformed to fit assumptions about homogeneity of multivariate dispersions (Anderson 2017). To obtain pairwise comparisons between communities, pairwise PERMANOVAs were performed between all Treatment × Time category combinations followed by correcting *p*‐values using the fdr method (Benjamini and Hochberg 1995). We used the *R* ^2^ value provided by the PERMANOVA as the effect size (ES). To provide a graphic summary of the results obtained from pairwise PERMANOVAs, we plotted the first‐axis values of non‐metric multidimensional scaling (NMDS) for each Treatment × Time interaction category in a boxplot. The letters in these plots represent the post hoc analysis results from the PERMANOVAs.

## 3 Results

### 3.1 Periphyton Accrual Over Time Between Treatments

Of the 19 response variables, 18 differed significantly across streams, and many variables significantly varied between treatments and sampling times (Table 2).

**TABLE 2:** Linear mixed model results for periphyton responses (data from all streams combined).

Treatment levels	Response variable	Stream			Stream/treatment			Stream/time			Stream/treatment × time		
		*p*	Sig.	ES	*p*	Sig.	ES	*p*	Sig.	ES	*p*	Sig.	ES
CT, PC	Biomass	0.264		0.01	0.036	**	0.34	0.053		0.05	0.335		0.01
	Cell density	0.002	**	0.21	0.001	**	0.39	0.509		0.07	0.073		0.09
	Taxa Richness	< 0.001	***	0.47	0.388		0.03	0.023	**	0.24	0.251		0.02
	Div‐Cyanobacteria, %	< 0.001	***	0.76	0.038	**	0.24	0.037	**	0.18	0.508		0.00
	Div‐Diatom, %	< 0.001	***	0.62	0.07	*	0.26	0.021	**	0.17	0.225		0.03
	Div‐Green algae, %	< 0.001	***	0.47	0.045		0.07	< 0.001	***	0.21	—	—	—
	Lf‐Colonial, %	< 0.001	***	0.59	0.554		0.01	0.346		0.01	0.057		0.17
	Lf‐Filamentous, %	< 0.001	***	0.64	0.032	**	0.11	0.749		0.24	0.030	**	0.13
	Lf‐Flagellate, %	< 0.001	***	0.59	0.01	**	0.20	0.045	*	0.14	0.278		0.01
	Lf‐Unicellular, %	< 0.001	***	0.74	0.026	**	0.28	< 0.001	***	0.52	0.68		0.00
	Att‐High, %	< 0.001	***	0.58	0.344		0.07	0.026	**	0.14	0.238		0.02
	Att‐Low, %	< 0.001	***	0.72	0.054		0.07	0.061		0.06	0.496		0.00
	Att‐Medium, %	< 0.001	***	0.75	0.128		0.05	0.217		0.06	0.986		0.00
	Mot‐Non motile, %	< 0.001	***	0.64	0.002	**	0.43	0.108		0.01	0.337		0.01
	Mot‐Drift, %	< 0.001	***	0.63	0.008	**	0.12	0.025		0.03	0.095		0.05
	Mot‐Gliding, %	0.022	**	0.10	0.666		0.00	0.022	**	0.17	0.126		0.05
CT, PT, DW	Community assemblage	0.001	***	0.27	0.001		0.06	0.001		0.09	0.049		0.03
	BP Dominance index	< 0.001	—	—	0.019	—	—	< 0.001	—	—	0.335	—	—
	Hill‐Shannon index	< 0.001	***	0.45	0.945		0.00	0.364		0.08	0.881		0.00

*Note:* Results for each predictor included *p*‐value and effect size (ES), significance level (*p* < 0.01, ES ≥ 0.1 = ***; *p* < 0.05, ES ≥ 0.1 = **; *p* < 0.10, ES ≥ 0.2 = *), and effect size (ES).

Abbreviations: att, Attachment category; CT, Control tiles; div, Algal division category; DW, drift‐water samples; lf, Life form category; mot, Motility category; PC, Plastic‐cover tiles.

**FIGURE 2:**
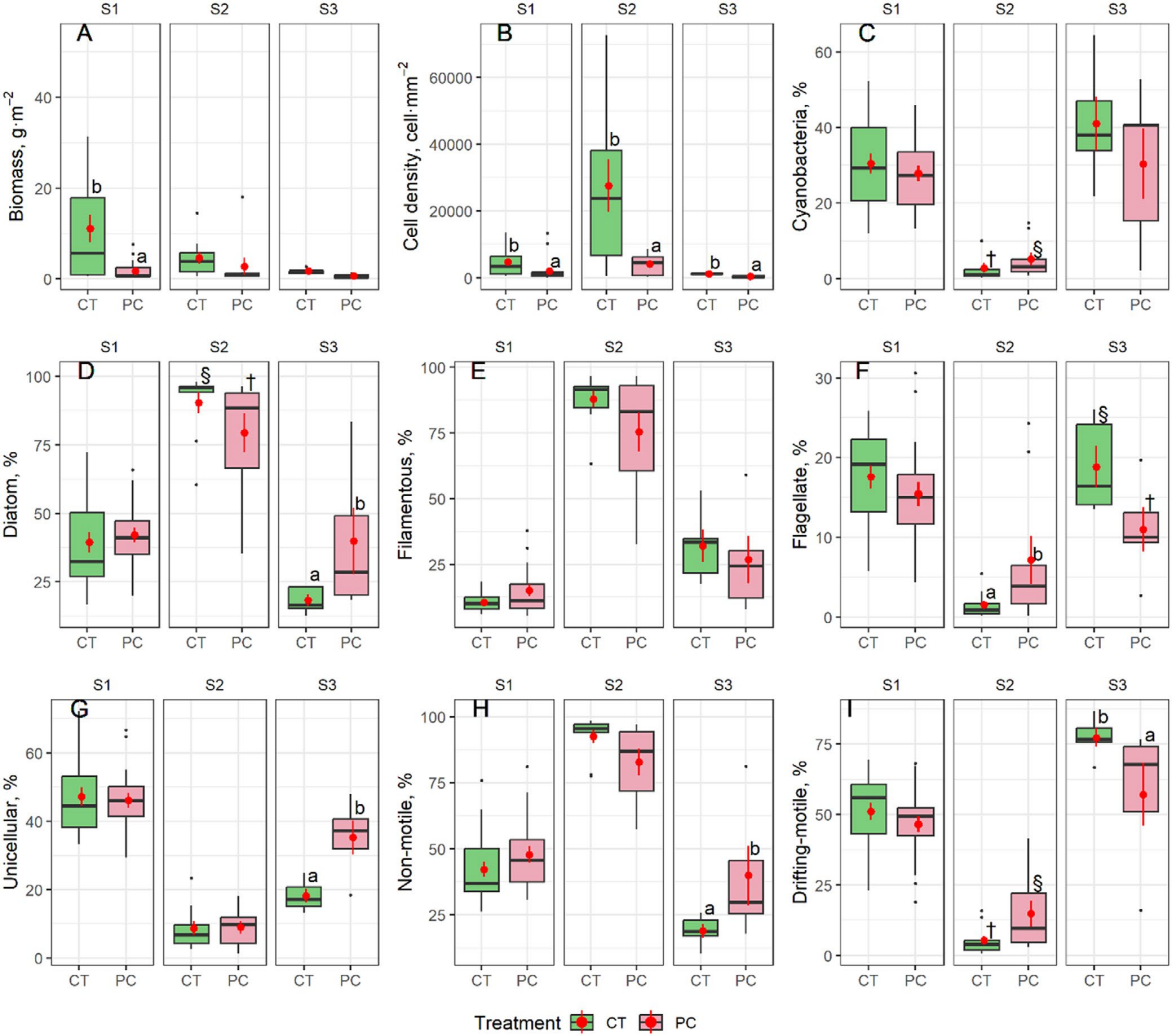
Boxplots illustrating periphyton responses in relation to Treatment and sampled stream. All plotted responses showed a significant Treatment effect in the LMMs (see Table 2). Treatments: CT, control tiles; PT, plastic‐cover tiles. Letters (a, b) indicate significant differences (*p* < 0.05) for post hoc tests, and symbols (†, §) indicate near‐significant differences (*p* < 0.10). Response variables: Biomass (A), cell density (B), cyanobacteria (%, C), diatoms (%, D), filamentous (%, E), flagellates (%, F), unicellular taxa (%, G), non‐motile taxa (%, H), and drift‐motile taxa (%, I). Point ranges in each boxplot represent the data average (point) and standard error (line).

**FIGURE 3:**
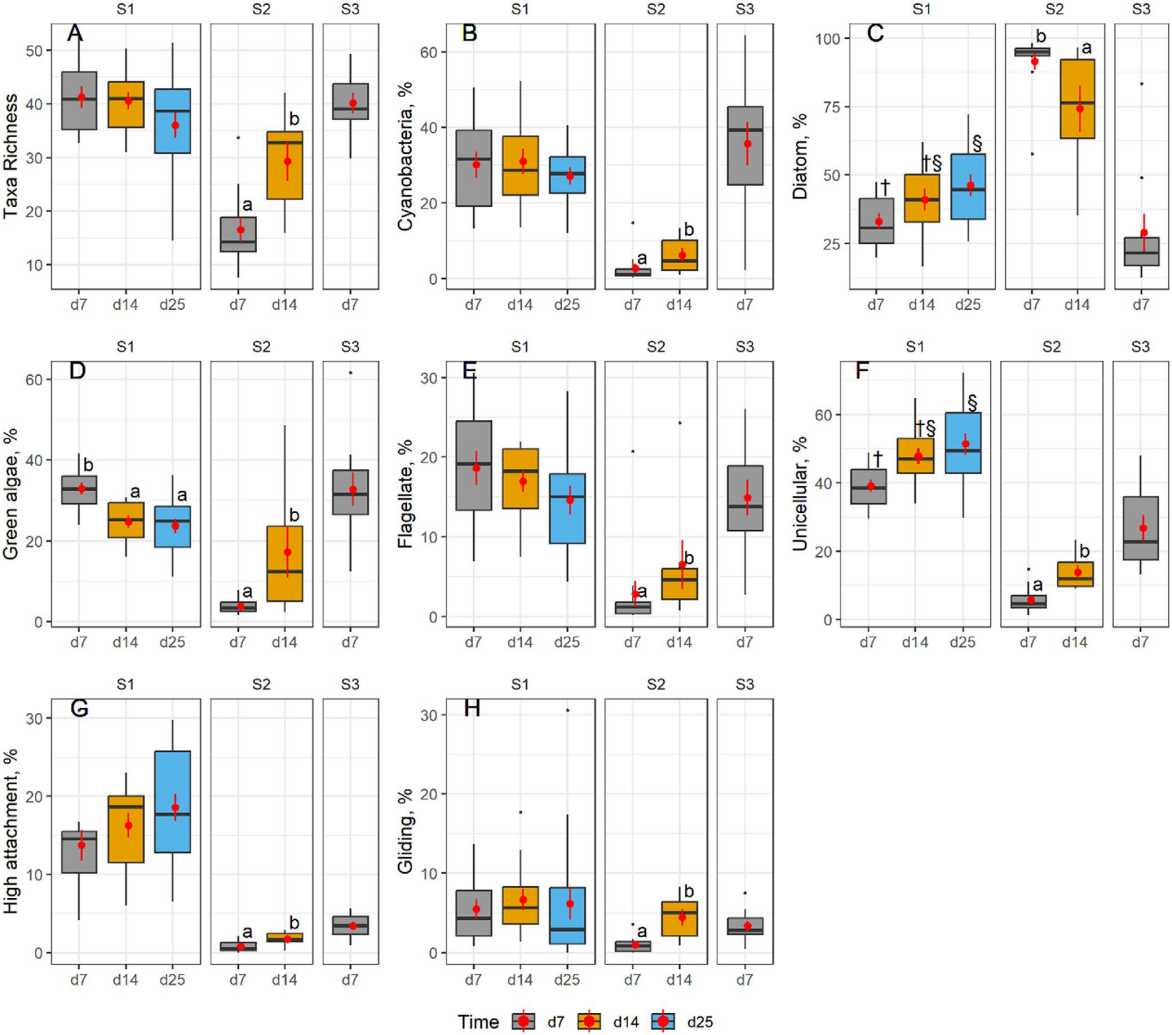
Boxplots illustrating periphyton responses in relation to sampling time and sampled stream. All plotted responses showed significant changes with time in the LMMs (see Table 2). Treatments: CT, control tiles; PT, plastic‐cover tiles. Letters (a, b) indicate significant differences (*p* < 0.05) for post hoc tests, and symbols (†, §) indicate near‐significant differences (*p* < 0.10). Response variables: Richness (A), cyanobacteria (%, B), diatoms (%, C), green algae (%, D), flagellates (%, E), unicellular taxa (%, F), high attached taxa (%, G), gliding taxa (%, H). Point ranges in each boxplot represent the data average (point) and standard error (line).

Overall, biomass and cell density were both lower on plastic‐cover tiles than on control tiles (Table 2, Figure 2A,B). Post hoc tests indicated that this difference was significant only in S1 for biomass but in all three streams for cell density. Despite this variation across streams, mean cell density and biomass on control tiles were 1.5–6 times higher than on plastic‐cover tiles. Richness only differed between sampling dates overall (Table 2), and post hoc analysis revealed this result was mainly based on significant differences between Day 7 and Day 14 in S2 (Figure 3A).

### 3.2 Functional Guild Differences Between Treatments

Periphyton trait metrics showed significant patterns either for treatment or time in most of the LMMs, except for colonial, low attachment, and medium attachment taxa (Table 2). Relative abundances of cyanobacteria, flagellates and drifting‐motile algae tended to be higher on plastic‐cover tiles in S2 but lower in S3 (Figure 2C,F,I). In contrast, diatoms and non‐motile taxa were rarer on plastic‐cover tiles in S2 but more prevalent in S3 (Figure 2D,H). Further, Unicellular taxa increased on plastic‐cover tiles in S3 (Figure 2G). Filamentous taxa showed no significant differences in post hoc tests, yet lower filamentous density was observed on plastic‐cover tiles in S2 (Figure 2E). The fewest differences between plastic‐cover and control tiles were found in S1, and the periphyton response patterns detected in S2 were generally the opposite of those in S3.

Differences across sampling days varied strongly between streams. Most of the guild categories increased from Day 7 to Day 14 in S2, such as cyanobacteria, green algae, flagellates, unicellular, high‐attachment, and gliding taxa (Figure 3B,D–H), while diatoms decreased (Figure 3C). Diatoms and unicellular taxa increased on Days 14 and 25 in S1 (Figure 3C,F), whereas green algae taxa decreased (Figure 3D). For unicellular taxa (Figure 3F), S1 and S2 showed similar patterns and an increase with time, but for green algae and diatoms (Figure 3C,D), S1 and S2 showed contrasting patterns. In S3, the values observed on Day 7 were more similar to those in S1 than in S2, except for high‐attachment taxa. Because of the general lack of significant Treatment × Time interactions (only one significant interaction for filamentous taxa), this pattern was not explored any further.

### 3.3 Comparison of Periphyton Community to Drift‐Water Samples

The PERMANOVA results for community assemblage, Hill‐Shannon index, and BP dominance index (Table 2) detected significant differences between sampling sites with large effect sizes. These differences were determined by strong differences in community composition and structure between sites due to a bloom of the diatom *Odontidium mesodon* observed in S2, whereas in S1 and S2 no taxon exhibited a clear density dominance. Yet, treatment, time and treatment × time interaction did not show as strong results.

**FIGURE 4:**
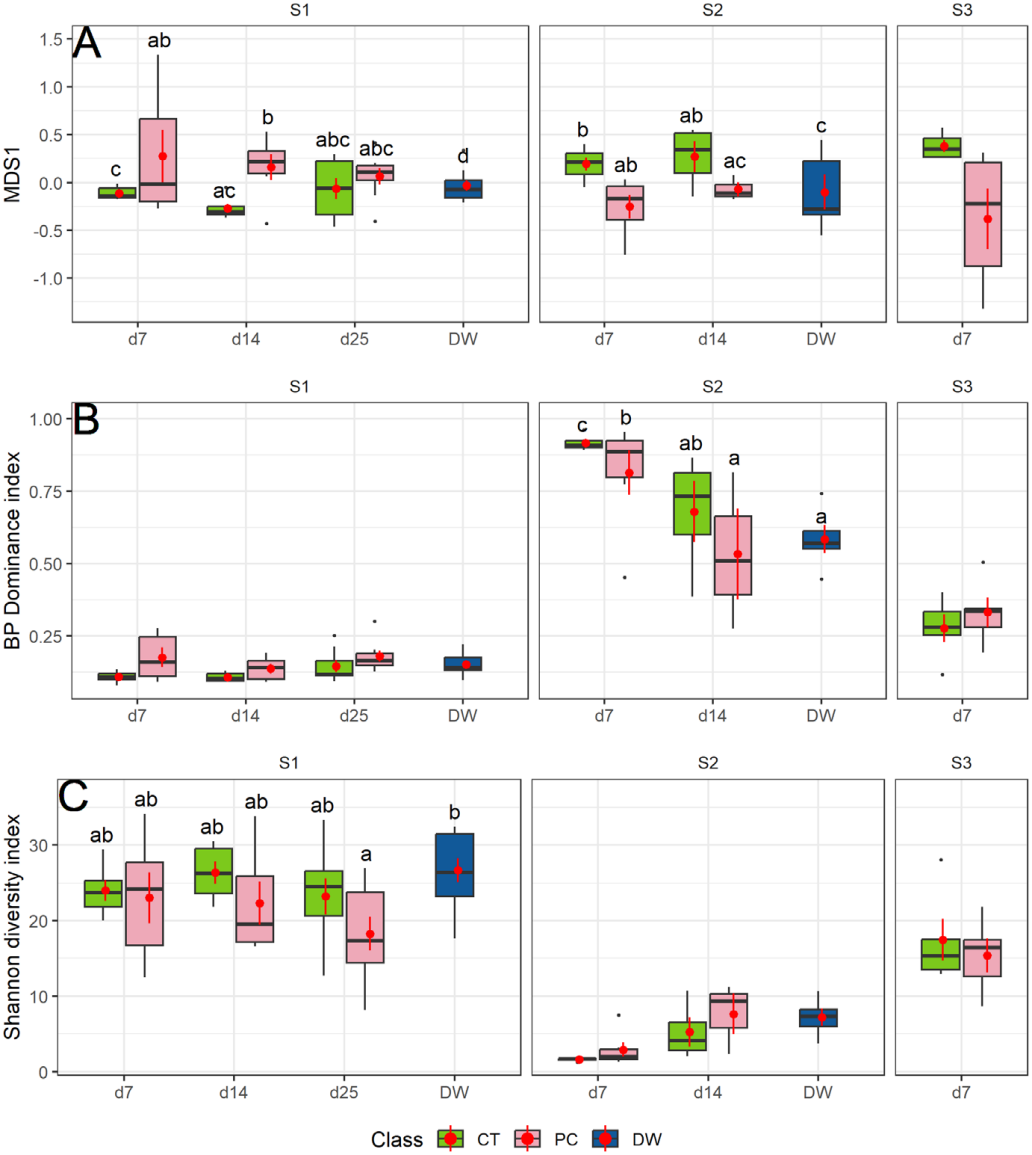
Boxplots illustrating periphyton community responses in relation to experimental treatments, sampling time and sampled sites. Treatments: CT, control tiles; DW, drift water; PT, plastic‐cover tiles. Letters (a, b, c) indicate significant differences (*p* < 0.05) for post hoc tests. Values plotted from the first axis of the non‐metric multidimensional scaling model (NDMS) (A), while post hoc values were obtained from pairwise PERMANOVA (for more details see Section 2). Berger‐Parker Dominance index (B). Shannon Diversity index corrected by sample size with iNEXT (Hsieh, Ma, and Chao 2016) (C). Point ranges in each boxplot represent the data average (point) and standard error (line).

In S1, DW samples exhibited a unique community significantly different from the remaining sample categories (Figure 4A). These DW samples also showed a higher BP Dominance index (Figure 4B) and Hill‐Shannon diversity index (Figure 4C) than periphyton samples, although this pattern was significant only for diversity. Community assemblage (Figure 4A) became more similar with time between control and plastic‐cover samples. The BP Dominance index increased slightly with time in both treatments, while plastic‐cover tiles generally exhibited a higher dominance index. Diversity tended to be constant across time and was slightly higher on control than on plastic‐cover tiles.

PERMANOVA results for S2 (Figure 4A) revealed similar patterns as in S1, with DW samples showing a community that differed from the periphyton community, although not with higher diversity in S2 (Figure 4C). In S2, no clear community patterns were observed with time; however, the Day 7‐Control community differed significantly from the Day 14‐plastic‐cover community. Dominance decreased over time, while diversity increased over time for both treatments (Figure 4B,C), although this pattern was significant only for BP Dominance. Finally, the post hoc analysis from the PERMANOVA for S3 showed no significant difference between plastic‐cover tiles and control tiles on Day 7; however, these tile categories were separated in the NMDS analysis due to a higher density of the green alga *Coelastrum* spp. on plastic‐cover tiles. Dominance and diversity indices did not change between treatments for S3 (Figure 4B,C).

## 4 Discussion

Our experiment investigated local periphyton colonisation versus upstream colonisation at the reach scale and represents, to our knowledge, the first study dedicated to disentangling the role of both propagule sources for periphyton community dynamics in running waters. Biomass and cell density were between 1.5 and 6 times lower on plastic‐cover samples than in control samples, suggesting that colonisation from nearby streambed patches was a key source of propagules and that slow colonisation rate impacts periphyton density. These results support our first hypothesis that plastic‐cover tiles should develop biofilms more slowly. Concerning our hypothesis 2 regarding the prevalence of taxon guilds with high dispersal capabilities, our findings were inconsistent across the three studied streams. For example, relative abundances of flagellates and drifting‐motile taxa on plastic‐cover tiles were higher than in controls (as predicted in H2) in Stream 2, but lower in Stream 3 and similar in Stream 1. Periphyton community composition on plastic‐cover tiles did not match the drift‐water community, contrary to our hypothesis 3, suggesting that stochasticity and habitat selection were interacting with dispersal processes during colonisation of the isolated plastic‐cover tiles.

### 4.1 Periphyton Colonisation Over Time

Our main finding was that the accrued periphyton cell density and biomass were 1.5–6 times higher when the experimental tile substrata were in direct contact with the streambed surface. While biomass consistently tended to be higher on control tiles, this pattern was significant only in Stream 1, whereas cell density showed significant differences across tile types in all three studied streams. We observed no further increase of cell density or biomass on Days 14 or 25, indicating that further biofilm accrual due to autochthonous primary production was very slow after Day 7. This result also implies that control tiles did not generate biofilm at a higher rate than plastic‐cover tiles, suggesting that the recorded differences in periphyton density standing stocks were determined by colonisation from elsewhere rather than by in situ (autochthonous) primary production on the tiles. Studies focusing on periphyton biomass accrual after floods observed a fast biomass recovery (within 15–40 days) in most cases (Matthaei, Guggelberger, and Huber 2003; Peterson et al. 1994; Schneck et al. 2017). By contrast, in our experiment, plastic‐cover samples did not reach a cell density or biomass similar to control samples even after 25 days, supporting the interpretation that our results did not depend on in situ production of periphyton.

Instead, we propose that the habitat around the control tiles determined their consistently higher cell density and that control tiles were exposed to higher periphyton immigration rates. More specifically, we suggest that a source‐sink dynamic may explain our findings for control tiles. Immigration of surplus individuals produced in nearby source populations (the streambed immediately surrounding the tiles) colonised the control tiles and increased their cell density. The source‐sink dynamic is a population‐level process that, when applied at the metacommunity level, leads to a mass effect metacommunity dynamic (Loreau and Mouquet 1999). In stream periphyton, it has previously been observed that habitat heterogeneity can increase taxon diversity and biofilm productivity by providing optimal habitats for more species (Besemer et al. 2009; Osório et al. 2019). Moreover, periphyton biofilm mats play a dual role in periphyton community dynamics by also providing more habitat heterogeneity with different strata inside these biofilms (Passy 2008, 2017). We suggest that habitat heterogeneity in the streambed around our control tiles produced suitable conditions for an individual reproduction surplus, thus creating an ideal scenario for a mass effect dynamic and increasing immigration rates into our control tile communities.

The idea of migration being an important periphyton metacommunity driver has been previously suggested in biofilm modelling studies at the microhabitat scale (Hödl et al. 2014; Woodcock et al. 2013), observed in in‐stream experiments (McCormick 1991; Peterson 1996a), and suggested by previous theoretical frameworks at the reach scale (Heino et al. 2015). Moreover, previous survey‐based studies at the catchment scale have observed that mass effect processes can affect diatom communities in running waters (Bottin et al. 2016; Leboucher et al. 2020). If we add our results showing high connectivity at the reach scale to the previous evidence for high connectivity at the catchment scale, we can suggest that periphyton communities are connected by propagules at various spatial scales. Previous experimental assessments of propagule sources originated from studies of periphyton recolonization in intermittent streams following drought (Robson 2000; Robson et al. 2008; Robson and Matthews 2004). These studies recognised the role of drifting and local propagules as potential propagule sources after flow resumption. In our case, we aimed for a characterisation of metacommunity processes under stable flow conditions, applying an experimental approach to streams with typically permanent flow, extending the scope of this idea and identifying drifting algae as a secondary source of propagules after local sources.

Differences between plastic‐cover tiles and control tiles were consistent across all streams. However, the loss of samples from Day 14 and Day 25 in Stream 3 and Day 25 in Stream 2 limited our ability to extrapolate our results regarding successional processes. Consequently, our confidence in the results from Day 25 is greatly reduced compared to Day 7. Considering H2 and H3, we expected that the community developing on plastic‐cover tiles would differ from communities on control tiles, ultimately leading to a completely new community by Day 25 due to differences in the main colonisers. In contrast, the community observed in plastic‐cover samples differed from that in control samples at all sites on Day 7 but tended to become more similar over time in Streams 1 and 2. This shift suggests that while communities in plastic‐cover samples on Day 7 were primarily determined by colonisers, by Day 25, habitat selection became more relevant for driving community structure. These results support the idea that environmental selection processes, while secondary, often become more important over time (Besemer et al. 2009; Hödl et al. 2014; Larson and Passy 2012). However, the lack of data from Streams 2 and 3 on Day 25 makes further discussion difficult. Stream 3 had the greatest morphological variability, but this was where we lost the most samples. Additionally, the experiment required fairly homogeneous, moderate hydraulic conditions at our study reaches. Thus, the narrow range of depth and velocity across our study reaches, combined with the lack of replicates from Streams 2 and 3, may have limited the role of environmental constraints over time in our study.

### 4.2 Functional Guild Responses

Our results about the different evaluated periphyton guilds were highly inconsistent across the three studied streams and can therefore be understood only in their unique context. The principal differences between our sites are that the reach in Stream 1 was bordered by dense riparian forest, whereas the reach in Stream 2 was surrounded by tussock grassland, and the reach in Stream 3 was surrounded by exotic pasture grazed by cattle. Most likely, the periphyton community in Stream 1 was strongly determined by its low productivity due to a general lack of sunlight. In this stream, neither richness, cell density, nor biomass changed with time, suggesting slow biofilm production. The most important changes recorded with time were a decrease in green algae and an increase in unicellular and diatom taxa. These changes suggest that in Stream 1, fast‐colonising taxa adapted to productive habitats, such as green algae colonised our tiles by Day 7, whereas by Day 25 the tile periphyton community was dominated by slower‐colonising taxa adapted to low‐productivity habitats as unicellular‐diatoms (Biggs 1996). In this stream, the clearest pattern observed between treatments was that motile and flagellate taxa tended to exhibit lower densities in plastic‐cover than in control samples on all sampling days. In this low‐productivity context, control tiles probably represented better habitats for motile and flagellate taxa due to their thicker biofilm mats, with lower shear stress and more diverse and complex functional diversity (Depetris et al. 2022; Larned et al. 2011).

The non‐shaded site in Stream 2 was dominated by *Odontidium mesodon*, a chain‐forming diatom classified as “filamentous”. The patterns detected in Stream 2 for Days 7 and 14 match those expected for mature biofilms. For example, no changes in biomass and cell density occurred between Day 7 and 14, suggesting that these biofilms quickly reached their productivity asymptote (Larson and Passy 2012; Peterson et al. 1994). At the same time, richness, green algae, cyanobacteria, flagellate, unicellular, high‐attachment, and gliding taxa all increased by Day 14, whereas diatoms decreased by Day 14. We suggest that all these results are related to the higher diversity observed on Day 14, when the relative abundance of the dominant diatom *Odontidium mesodon* decreased, while all remaining guilds increased. An increase in diversity in mature biofilms is expected based on the literature, where dominant taxa generate a resource‐depleted biofilm and tolerant species still persist (Passy 2008, 2017; Peterson 1996a). In our experiment, flagellates and motile taxa were more prevalent in plastic‐cover samples than in controls in Stream 2, contrary to the pattern in Stream 1. Due to the specific context of Stream 2, we suggest that this result was determined by motile taxa reaching open habitats available for colonisation, an idea proposed in several previous studies (Bondoc et al. 2016; Passy 2007; Serôdio et al. 2023).

The site in Stream 3 produced the least information because of the drought in this stream. Despite being a non‐shaded stream, Stream 3 showed similar cell density and richness to the shaded Stream 1. We do not have repeated sampling that would allow determination of the succession stage of the biofilms in Stream 3, yet the low cell density and biomass on Day 7 suggest a low biofilm production in this stream. Motile and flagellate taxa were less common on plastic‐cover tiles than on controls, as in Stream 1. Nevertheless, the higher prevalences of non‐motile, unicellular, and diatoms on plastic‐cover tiles compared to controls are challenging to explain. These guilds are closely related, with all three comprising non‐filamentous diatoms with low motility. Such diatoms need high shear stresses to be removed from streambed substrata (Biggs, Stevenson, and Lowe 1998; Holland et al. 2004; Steinman 1996), and high shear stress did not occur during our study. However, on Day 7 we noticed that the plastic‐cover treatment in Stream 3 had an important inflow of water from the hyporheic zone; therefore, this groundwater inflow could represent a relevant propagule source, perhaps providing a different community of colonists (Land and Peters 2023). Unfortunately, the lack of drift periphyton samples from Stream 3 prevented us from comparing drift communities to tile communities and further interpreting our findings.

Periphyton community assembly in our study was modulated by an interaction between functional guilds, biofilm succession stage, and river productivity, as previously proposed (Lange, Townsend, and Matthaei 2016; Law, Elliott, and Thackeray 2014; Passy 2007; Passy and Larson 2011). Despite the particularities of all three streams, motile and flagellate taxa consistently exhibited an important role on our tile substrata. This finding suggests that these guilds can reach optimal habitats at the reach scale, allowing them to increase their prevalence on isolated substrata located ~15 m from the closest periphyton‐covered bed surfaces in Stream 2, or to avoid low‐quality habitats in Stream 1. We did not find any evidence supporting the idea that filamentous algae exhibit higher dispersal capabilities because of shear‐drag removal (Biggs, Stevenson, and Lowe 1998; Lange, Townsend, and Matthaei 2016), nor for the idea that diatoms, cyanobacteria, or unicellular taxa should be early colonisers (Biggs, Stevenson, and Lowe 1998). Further research is needed to provide more evidence for the patterns we observed, but based on our findings we conclude that motile taxa possess advantages when colonising optimal habitats at the reach scale and that these advantages affect community assemblages. However, for the rest of the functional guilds, we found inconclusive results regarding their dispersal patterns.

### 4.3 Tile Community Versus Drift‐Water Community

Our main question about the periphyton drifting community was whether it can predict the plastic‐cover tile community because the latter should be derived mainly from upstream colonisation. In fact, drift samples tended to exhibit higher richness and Shannon diversity than both tile treatments (control, plastic‐cover), with this pattern being significant in Stream 1 and a weaker trend in Stream 2. Moreover, the BP Dominance index suggested the most homogeneous density patterns for the drift communities, supporting the idea that these communities are more even. This pattern has also been detected in previous studies in headwater streams (Besemer et al. 2012, 2013), which proposed that these results were explained by the different headwater lateral surface inflows, principally from terrestrial microbial communities. Moreover, due to the significant differences between the drift‐water community and both tile periphyton communities, our results suggest the former did not directly determine the plastic‐cover tile communities. Similarly, previous studies concluded that immigration from the periphyton drift community alone could not successfully explain the composition of early‐successional stream biofilm communities (McCormick 1991; Peterson 1996a; Stevenson 1984; Woodcock et al. 2013). Consequently, these authors argued that it is necessary to include emigration and competition to obtain a more complete picture. Additionally, studies of periphyton recolonization after flow resumption following drought suggested that the roles of drifting and local communities as propagule sources could vary between streams, but without drawing any conclusions regarding which factors might determine the respective roles of these two propagule sources (Robson et al. 2008).

Temporal dynamics have been detected in drifting communities on a diurnal scale, with peak densities for specific taxa at particular times of the day (Peterson 1996a; Stevenson and Peterson 1991). For example, Peterson (1996a) collected periphyton drift‐community samples every 3 h for 36 h on two occasions in summer. Peterson found that, while some taxa showed a relatively constant density of drift propagules, other taxa exhibited daily peaks of drift density, some of them at midday and others after sunset. These peaks were produced by attached and non‐attached diatoms, colonial green algae, and cyanobacteria. Periphyton communities exhibit a successional process, observable in a span of days to weeks, and this succession is shaped by deterministic and stochastically processes and periphyton functional guilds (Matthaei, Guggelberger, and Huber 2003; Passy 2017; Peterson 1996b). However, based on the findings of Peterson Peterson (1996a, 1996b), it appears that at the taxon level, periphyton drift community composition is determined by specific dispersal events which occur within a few hours, presumably driven by upstream periphyton productivity. Consequently, the relationship between the benthic and drifting periphyton communities is still poorly understood, and our capability to describe periphyton community dynamics could be improved by including hourly replicates of drift‐water samples.

## 5 Conclusions

As far as we know, this is the first study worldwide that empirically shows that periphyton dispersal from the local streambed can be more important than upstream colonisation for determining periphyton metacommunity assemblage in running waters. While tile substrata exclusively colonised by the drift periphyton community were still able to generate biofilms, the low biomass and cell density of these biofilms strongly suggest that periphyton communities depend on colonisation from the nearby streambed to obtain higher levels of biomass and density. Our findings regarding the relative abundances of functional periphyton guilds were driven by differences across streams. Motile taxa and flagellates fulfilled theoretical expectations by reaching higher prevalences in more suitable habitats, highlighting their ability to overcome the dispersal barrier imposed by 25 m of plastic sheet cover. However, the remaining guild abundance patterns detected were inconsistent with the theoretical expectations. Moreover, the drift periphyton community was not strongly related to the plastic‐cover or control benthic periphyton communities. In related future research, we recommend conducting more extensive drift sampling, for example, to detect hourly dispersal patterns in the drift community, to help develop a better understanding of the relationship between the drift and benthic periphyton communities.

## Author Contributions

**Daniel Zamorano:** conceptualization (equal), data curation (lead), formal analysis (lead), investigation (lead), methodology (equal), project administration (lead), visualization (lead), writing – original draft (equal), writing – review and editing (equal). **Travis Ingram:** conceptualization (supporting), data curation (supporting), investigation (supporting), methodology (supporting), writing – original draft (equal), writing – review and editing (equal). **Christoph D. Matthaei:** conceptualization (equal), data curation (supporting), investigation (supporting), methodology (equal), supervision (lead), writing – original draft (equal), writing – review and editing (equal).

## Conflicts of Interest

The authors declare no conflicts of interest.

## Data Availability

The raw data of this study, including environmental variables and taxonomic sheet, plus the R code with all models and graphs, were included as online supplement at the following link: https://figshare.com/s/1a56a775295394d37590. Upon acceptance, data and code will be provided via the following link: https://doi.org/10.6084/m9.figshare.19882087.
